# Efficacy of hyaluronic acid and butyroyl glutathione in the management of glaucoma-related ocular surface disease: a prospective, interventional, double-blind, cross-over post market study

**DOI:** 10.3389/fphar.2026.1780815

**Published:** 2026-06-05

**Authors:** Gabriele Gallo Afflitto, Marialaura Fanelli, Vita Petrone, Rossella Chirico, Francesca Ceccarelli, Alessio Martucci, Antonella Minutolo, Francesco Aiello, Enrico Garaci, Claudia Matteucci, Carlo Nucci

**Affiliations:** 1 Ophthalmology Unit, Department of Experimental Medicine, University of Rome Tor Vergata, Rome, Italy; 2 Moorfields Eye Hospital NHS Foundation Trust, London, United Kingdom; 3 Microbiology and Clinical Microbiology, Department of Experimental Medicine, University of Rome Tor Vergata, Rome, Italy; 4 Casa di cura San Raffaele, Sulmona, L’Aquila, Italy

**Keywords:** cytokines, dry eye syndromes, glaucoma, glutathione, lubricant eye drops, wound healing

## Abstract

**Introduction:**

To evaluate the efficacy of a novel eye drop formulation containing hyaluronic acid (0.2%) and butyroyl-glutathione (GSHC4, 0.4%) in glaucoma-associated ocular surface disease (G-OSD), and to explore putative mechanisms of action through in vitro assays of corneal epithelial wound healing and cytokine modulation under basal, inflammatory, and oxidative stress conditions.

**Methods:**

In this preliminary, hypothesis-generating, prospective, double-blind, cross-over study, 16 patients with glaucoma or ocular hypertension and coexisting dry eye symptoms were randomized to receive either HA alone or HA + GSH-C4 for 4 weeks, separated by a 1-week washout. Clinical endpoints included the Ocular Surface Disease Index (OSDI), FACES scale, tear film break-up time (TFBUT), Schirmer test, and NEI fluorescein staining. Tear cytokines were quantified at multiple timepoints. In vitro, human corneal epithelial cells underwent scratch-wound assays and cytokine profiling.

**Results:**

Fifteen of sixteen patients completed the study. HA + GSH-C4 significantly improved OSDI (p < 0.001), FACES scores (p = 0.012), TFBUT (p < 0.001), and NEI-SS (p < 0.001) compared with HA alone. No treatment-related adverse events were observed. Tear cytokine analysis revealed a reversible treatment-specific elevation of selected mediators (e.g., IFN-γ, IL-12p70) consistent with a controlled pro-repair response. The HA + GSH-C4 accelerated epithelial wound closure and selectively suppressed MCP-1 across all tested conditions.

**Discussion:**

The HA + GSH-C4 formulation improved G-OSD symptoms and ocular surface stability, accompanied by a dynamic cytokine response in tears. These findings support its clinical utility and suggest a dual mechanism involving barrier restoration and modulation of ocular surface immune responses.

## Graphical Abstract

**Figure:**
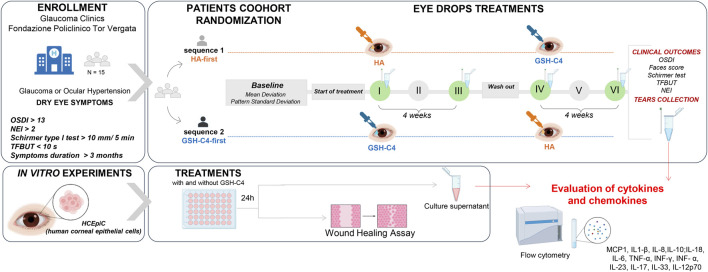

## 1 Introduction

Glaucoma refers to a heterogeneous group of chronic, progressive optic neuropathies characterized by the irreversible loss of retinal ganglion cells, leading to thinning of the retinal nerve fiber layer and subsequent visual field loss (Gallo et al., 2022; Gallo Afflitto et al., 2023; Gallo Afflitto et al., 2025). It is the leading cause of irreversible blindness worldwide, with prevalence rising significantly with age and varying across different ethnic groups (Gallo et al., 2022; Gallo and Swaminathan, 2024). Despite the range of therapeutic strategies available, current treatments can only slow disease progression (Gallo Afflitto et al., 2025; Gazzard et al., 2019; Gallo and Swaminathan, 2023; Gallo et al., 2025). Among these options, topical hypotensive agents remain a first-choice treatment (Khawaja et al., 2020; Shaheen et al., 2024). However, the active ingredients, preservatives, and excipients in these formulations may collectively damage cellular structures, ultimately causing alterations and instability of the tear film (Nijm et al., 2020).

These factors contribute to the development of glaucoma-related ocular surface disease (G-OSD), a condition that often goes underdiagnosed but is estimated to affect 40%–59% of glaucoma patients worldwide (Nijm et al., 2020). Recently, Sedlak et al. described that, in patients with ocular hypertension or glaucoma, the use of prostaglandin F2α analogues induces an inflammatory state accompanied by increased local oxidative stress, including a reduction in glutathione peroxidase activity (Sedlak et al., 2019). Similarly, Tovar et al. reported that eyes receiving topical hypotensive medications exhibited significantly higher concentrations of caspase-1—a downstream mediator of inflammasome activation involved in dry eye disease (DED)—than both DED-affected and control eyes, with a positive correlation between caspase-1 levels and the number of topical medications administered (Tovar et al., 2022). For DED, the first-line pharmacologic treatment typically involves preservative-free eye drops containing hyaluronic acid (HA) (Craig et al., 2017). While HA enhances tear hydration, it may not sufficiently interrupt the multifactorial cycle of G-OSD (Nijm et al., 2020; Craig et al., 2017).

Glutathione (GSH), a low molecular weight thiol-containing compound, is the most abundant biological antioxidant present in mammalian tear fluid (Gukasya et al., 2007). Several studies have suggested that GSH depletion is a key indicator of apoptosis and caspase activity, affecting both its catalytic function and proteolytic activation (Circu and Aw, 2012). Notably, a reduction in thiol-containing compounds has been reported on the ocular surface of patients treated with benzalkonium chloride-preserved antiglaucoma medications (Sedlak et al., 2021). To date, butyroyl glutathione (GSH-C4), a cell-permeable derivative of glutathione (GSH), has been investigated primarily in dermatologic and systemic settings, where it has demonstrated antioxidant and immunomodulatory properties (Limongi et al., 2019). In topical formulations for inflammatory skin conditions, GSH-C4 combined with HA has shown favorable efficacy and tolerability in preliminary clinical studies. Systemic studies in murine models of viral infection have further indicated that GSH-C4 enhances intracellular GSH levels and promotes a Th1-skewed immune response (Limongi et al., 2019; Campione et al., 2019). However, its use in ophthalmology—either *in vitro* or *in vivo*—has not yet been explored.

Given the multifactorial nature of glaucoma-associated ocular surface disease (G-OSD)—which involves chronic inflammation, oxidative stress, and tear film instability—developing novel therapies that target these intersecting pathways is of considerable clinical relevance. Therefore, the aim of this study was to evaluate the clinical efficacy of a new ophthalmic formulation containing HA (0.2%) and GSH-C4 (0.4%) in patients with G-OSD. In parallel, *in vitro* proof-of-concept experiments were conducted to elucidate putative mechanism of action of this formulation, focusing on wound healing and cytokine modulation under basal, inflammatory, and oxidative conditions.

## 2 Materials and methods

This study complied with all applicable national regulations, adhered to the principles outlined in the Declaration of Helsinki and the Health Insurance Portability and Accountability Act, and was approved by the Institutional Review Board at the University of Rome “Tor Vergata” (RS53.22). Subjects for the study were identified from the glaucoma clinics at the Fondazione Policlinico “Tor Vergata”. Written informed consent was obtained from each participant prior to their enrollment in the study. No modifications or deviations from the original protocol were documented.

### 2.1 Study design

In this prospective, double-blind, cross-over interventional study, patients were randomized in balanced blocks (1:1) into two groups: Sequence 1, HA-first, received the control product (containing only hyaluronic acid 0.2%) for 4 weeks, followed by a 1-week washout, and then the experimental product (hyaluronic acid 0.2% + butyroyl glutathione 0.4%) for 4 weeks. Conversely, Sequence 2, GSH-C4-first, received the experimental product for 4 weeks, followed by a 1-week washout, and then switched to the control product for a further 4 weeks.

**FIGURE 1:**
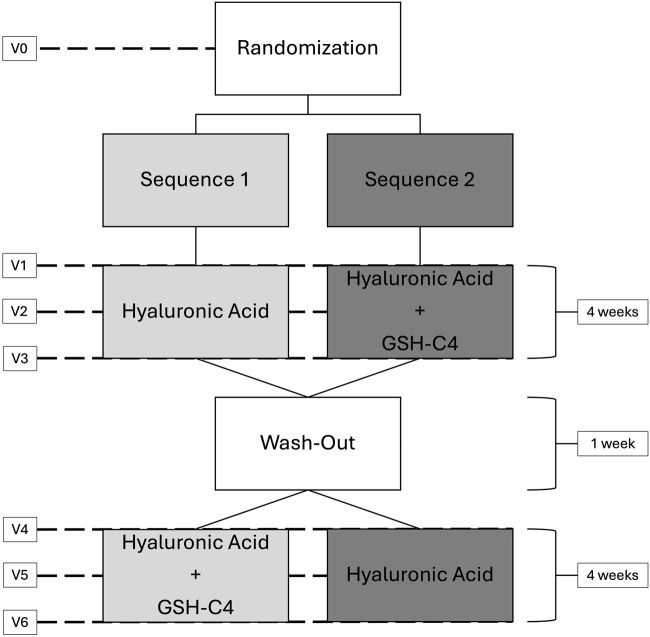
Study design and randomization flow. Patients were randomized (V0) in a 1:1 ratio to either Sequence 1 (Hyaluronic Acid followed by Hyaluronic Acid + GSH-C4) or Sequence 2 (Hyaluronic Acid + GSH-C4 followed by Hyaluronic Acid). Each treatment period lasted 4 weeks, separated by a 1-week wash-out phase. Clinical visits were performed at baseline (V0) and at each subsequent time point (V1–V6) as indicated.

Following randomization, enrolled patients entered a 9-week follow-up period, consisting of one 4-week treatment cycle with the experimental product and one 4-week cycle with the control product. A 1-week washout period from any preservative-free topical ocular lubricant preceded the start of the study and separated the two treatment cycles. Patients were evaluated every 2 weeks, according to the schedule depicted in Figure 1.

### 2.2 Inclusion and exclusion criteria

Patients of both sexes over the age of 18 with G-OSD were included in the study. Specifically, patients were required to have a diagnosis of glaucoma or ocular hypertension according to European Glaucoma Society criteria (European Glaucoma Society Terminology and Guidelines for Glaucoma, 2017), be undergoing topical hypotensive therapy that included at least one prostaglandin F2α analog, and have a diagnosis of dry eye based on the simultaneous presence of the following criteria: Ocular Surface Disease Index (OSDI) > 13, National Eye Institute fluorescein staining score >2, Schirmer test (type I) > 10 mm/5 min, symptom duration >3 months, and tear film break-up time (TFBUT) < 10 s. No restrictions were imposed on the severity of baseline glaucomatous visual field damage, intraocular pressure level, or the number of topical hypotensive agents used at baseline.

Patients were excluded if they had undergone any ocular surgery within the past 3 months; had previous corneal surgery; had an active or past corneal pathology (e.g., corneal ulcer, corneal dystrophy); were diagnosed with cicatricial conjunctivitis; had a history of mechanical or chemical corneal trauma; had an autoimmune disease (e.g., systemic lupus erythematosus, rheumatoid arthritis, Sjögren’s syndrome); wore contact lenses; had an allergy to any component of the study drug; or had participated in another clinical trial within the 3 months prior to the start of the study. Lack of capacity, compliance issues, or inability to self-administer topical eye drops were also considered exclusion criteria.

### 2.3 Randomization

Participants were randomized (1:1) using a pre-specified block randomization sequence (BABA–BAAB–AABB–ABBA), ensuring balanced allocation across treatment sequences. The randomization list was concealed and remained inaccessible to study participants, investigators, monitors, sponsors, and laboratory staff until database lock. Both the experimental and control formulations were packaged in identical cartons labeled only with protocol and kit numbers, preventing visual differentiation and preserving masking throughout the study.

### 2.4 Sample size calculation

The study was designed to include 16 eyes (8 in each group), providing a 95% statistical power to detect a significant difference between the hyaluronic acid + glutathione group and the hyaluronic acid-only group, assuming a 20% dispersion index and a two-sided α = 0.05 (Chow et al., 2017). The anticipated means and standard deviations used for this calculation were derived from tear TNF-α levels reported by Jee et al. (2014). Although TFBUT was designated as the primary clinical endpoint, it is known to be examiner-dependent and variable, which may limit reproducibility in small exploratory trials. For this reason, powering was performed on TNF-α due to the absence of prior ophthalmic data for GSH-C4. This choice underscores the exploratory, proof-of-concept nature of the trial: while cytokine modulation provided a feasible basis for estimating sample size, clinical endpoints were not definitively powered.

### 2.5 Study procedures

All tests and data collection were performed by a cornea specialist. Data were recorded on a structured form that included forced-choice entry criteria. Both clinical and patient-reported outcome measures (PROMs) were investigated. Among the former, fluorescein staining of the ocular surface was scored according to the recommendations of the National Eye Institute/Industry workshop on clinical trials for dry eyes (NEI-SS) (Lemp, 1995). Schirmer’s test type I (i.e., without anesthesia) and tear film break-up time (TFBUT) were used to assess tear film production and stability, respectively. Two PROMs were evaluated: the Ocular Surface Disease Index (OSDI) and a subjective visual scale (faces score), according to Noble et al. (2004) Adverse events were documented if present. Treatment compliance was assessed by measuring the remaining medication in the returned eye drop bottles at the end of each treatment period.

Tear samples were collected at visits V1, V3, V4, and V6 using Schirmer strips, as previously described in the literature (VanDerMeid et al., 2011). Schirmer strips were then placed in a 1.5 mL tube on ice. The tear samples were recovered by centrifugation at 13,000 RPM x 20′at 4 °C.

### 2.6 Analysis of cytokines in tears and HCEpiC cell cultures

To determine cytokine levels from recovered tears and supernatant from treated HCEpiC cells, the LEGENDplex™ Human Inflammation Panel 1 (13-plex) flow cytometry-based assay kit was used (BioLegend, San Diego, CA). This panel allowed the simultaneous quantification of 13 human cytokines and chemokines (IL-1β, IFN-α2, IFN-γ, TNF-α, MCP-1 (CCL2), IL-6, IL-8 (CXCL8), IL-10, IL-12p70, IL-17A, IL-18, IL-23, and IL-33). Detection of cytokine levels was performed as recommended by the manufacturer’s instructions. The samples were processed by CytoFLEX (Beckman Coulter) and CytExpert 2.3 software. Data were analysed using the standard curve detection and online software platform provided by the producer.

### 2.7 *In vitro* study and wound healing assay

Human Corneal Epithelial Cells (HCEpiC) isolated from human healthy cornea (Innoprot, Derio, Spain) were used to analyses the eye drop formulations *in vitro*. The cell line was cultured with Corneal Epithelial Cell Medium Kit in poly‐L‐lysine‐coated flasks (2 μg/cm^2^) with 5% (v/v) heat-inactivated fetal bovine serum (FBS). The cell line was maintained in an incubator at a temperature of 37 °C, in a humid atmosphere and in the presence of 5% CO_2_. Cell cultures were conducted under sterile conditions using a laminar flow hood. HCEpiC were treated with the two formulations used in human trial, containing hyaluronic acid 0.2% + Butyroyl glutathione 0.4% (or hyaluronic acid 0.2% only). To evaluate their capacity to promote cellular repair, a wound-healing assay was performed. In brief, 50,000 cells were seeded in 48-well polystyrene plates in the presence of 500 µL media. After 12 h of culture, an incision was made in the centre of the cell monolayer. Subsequently, treatments with eye drop preparations containing hylauronic acid with or without GSH-C4 (25 µL/well and 50 µL/well of eye drop products) was initiated both in the absence and presence of Lipopolysaccharide (LPS) (1 μg/mL) or hydrogen peroxide (H_2_O_2_) (25 µM) added to induce stress conditions. At the time of incision (0 h), after 24 and 48 h of incubation with treatments, three photos per well were acquired under an optical microscope (Olympus IX51 model) at a magnification of 4×. Cell migration was analysed by the ‘ImageJ’ software.

### 2.8 Statistical analysis

Statistical analyses were conducted in R (The R Project for Statistical Computing, Vienna, Austria) for clinical variables, and in SPSS software (IBM SPSS Statistics, Chicago, IL, USA; version 23.0) for preclinical data. Data distribution was initially assessed using the Shapiro–Wilk normality test. Rasch-adjusted scores were applied to the Faces scale to convert ordinal responses into interval-level measurements, according to the estimates proposed by Noble et al. (2004) Given the paired nature of the crossover design and the non-normal distribution of some variables, within-subject comparisons across treatment periods were performed using paired Wilcoxon Signed-Rank tests, with p-values adjusted for multiple comparisons using the Bonferroni–Holm method. In addition, within-sequence longitudinal changes from baseline were assessed using paired Wilcoxon Signed-Rank tests to capture temporal dynamics across visits. Summary statistics, reported as mean ± SD, were computed for each outcome under both the control formulation (i.e., Sequence 1 at V1, V2, V3; Sequence 2 at V4, V5, V6) and experimental formulation (i.e., Sequence 2 at V4, V5, V6; Sequence 1 at V1, V2, V3) conditions. Confirmatory analyses were carried out following the principles outlined by Wellek and Blettner (2012). A mixed-effects model was built to assess the presence of any carryover effects. This model included fixed effects for treatment condition, period, and treatment sequence (reflecting the order in which treatments were administered), along with a random intercept for each eye. The absence of a statistically significant sequence effect was interpreted as evidence against a substantial carryover effect. Pairwise associations between continuous variables were tested using Spearman’s correlation coefficient. ANOVA with Bonferroni post-hoc multiple comparison was performed to analyze variance and determine the significance of differences across treatment conditions. Statistically significant comparisons were considered at p < 0.05, with exact p-values reported; for highly significant findings, p < 0.001 is indicated. Statistically significant comparisons were considered at p < 0.05. All analyses were performed on a per-protocol (PP) dataset, excluding any instances of deviation from protocol for both *in vivo* and *in vitro* data.

## 3 Results

A total of 16 consecutive eyes from 16 patients were included in the study. One participant from Sequence 1 (i.e., HA-first) was excluded after reporting ocular side effects - specifically ocular irritation. It was subsequently discovered that these symptoms occurred while the participant was using control eye drops. Because this participant withdrew at V2, no subjective or objective data were available for analysis. This withdrawal was excluded from the PP dataset without any attrition bias, as our sample size calculation assumed a 20% dispersion rate.

Of the remaining 15 patients, 5 (33.3%) were female. The mean age of the entire cohort was 71.3 ± 9.6 years, with no statistically significant difference between Sequence 1 (HA-first) (68.1 ± 10.2 years) and Sequence 2 (GSH-C4-first) (74.5 ± 8.4 years) (p = 0.2898). Table 1 presents the clinical and demographic characteristics of the selected group at baseline (i.e., V0).

**TABLE 1:** Baseline (i.e., V0) clinical and demographic features of patients included in the analysis. No significant differences were observed between treatment sequences. Results are expressed as mean ± standard deviation (Abbreviations: dB = decibels; GSH-C4 = butyroyl glutathione; HA = hyaluronic acid).

Variable	Sequence 1 (HA first)	Sequence 2 (GSH-C4 first)	P^*^
Age (years)	74.3 ± 6.7	74.5 ± 8.9	0.289
No of antiglaucoma drops	2.0 ± 0.5	2.3 ± 0.7	0.907
Mean deviation (dB)	−7.1 ± 3.6	−9.5 ± 4.8	0.613
Pattern standard deviation (dB)	8.3 ± 4.8	7.8 ± 3.7	0.867
Ocular surface disease index	21 ± 8.7	23 ± 7.4	0.587
Faces (rasch) score	2.8 ± 0.6	2.6 ± 0.5	0.813
Schirmer test (mm/5 min)	11.5 ± 0.5	11.5 ± 0.6	>0.999
Tear film break up time (seconds)	6.0 ± 1.2	5.5 ± 1.3	0.967
National eye institute staining score	8.4 ± 2.1	8.5 ± 2.3	>0.999

^*^ Mann-Whitney U test.

### 3.1 Clinical outcome measures

**FIGURE 2:**
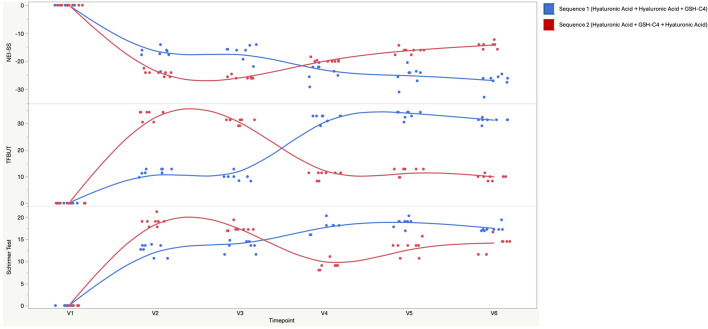
Baseline-normalized percentage change in outcome measures over time by treatment sequence. Shown are the National Eye Institute-Staining Score (NEI-SS, top), tear break-up time (TBUT, middle), and Schirmer test (bottom). Values are expressed as percentage change from baseline (V0). Blue = Sequence 1 (Hyaluronic Acid → Hyaluronic Acid + GSH-C4); red = Sequence 2 (Hyaluronic Acid + GSH-C4 → Hyaluronic Acid). A one-week wash-out occurred between V3 and V4. Negative change in NEI-SS indicates symptom improvement; positive change in Schirmer test and TBUT indicates enhanced tear function.

Table 2; Figure 2 indicate that, in the selected cohort, HA + GSH-C4 treatment did not produce a statistically significant overall effect on the Schirmer test (Control formulation: 12.35 ± 1.47 vs. Experimental formulation: 13.02 ± 0.84; p = 0.054). However, in Sequence 2 (i.e., GSH-C4-first) exhibited a significant median increase of 3 mm from baseline to the intermediate assessment (p = 0.0345).

**TABLE 2:** Clinical outcomes of the selected cohort under investigation. The Control formulation (i.e., HA-only) includes Sequence 1 (i.e., HA-first) during V1–V3 and Sequence 2 (i.e., GSH-C4-first) during V4–V6. The Experimental group (i.e., HA + GSH-C4) includes Sequence 1 during V4–V6 and Sequence 2 during V1–V3 (Abbreviations: GSH-C4 = butyroyl glutathione; HA = hyaluronic acid).

Variable	HA	HA + GSH-C4	P^§^	Adjusted P^*^	Sequence effect
Ocular surface disease index	25.26 ± 2.9	20.11 ± 2.35	<0.001	0.001	0.127
Faces (rasch) score	3.30 ± 0.86	2.38 ± 0.19	0.012	0.031	0.089
Schirmer test (mm/5 min)	12.35 ± 1.47	13.02 ± 0.84	0.054	0.068	0.154
Tear film break up time (seconds)	5.81 ± 0.35	7.31 ± 0.57	<0.001	0.001	0.241
National eye institute staining score	4.17 ± 0.34	3.81 ± 0.23	<0.001	0.001	0.392

^§^ Wilcoxon Signed‐Rank tests.

* Bonferroni–Holm method.

A statistically significant improvement was observed in both TFBUT (Control formulation: 5.81 ± 0.35 vs. Experimental formulation: 7.31 ± 0.57; p < 0.001) and the NEI-SS score (Control formulation: 4.17 ± 0.34 vs. Experimental formulation: 3.81 ± 0.23; p < 0.001) in eyes treated with HA + GSH-C4.

No carryover effect was detected for any of the analyzed clinical endpoints, as detailed in Table 2.

### 3.2 Patient-reported outcome measures (PROMs)

As summarized in Table 2, HA + GSH-C4 treatment was associated with a reduction in OSDI scores (control formulation: 25.26 ± 0.05 vs. experimental formulation: 20.11 ± 0.03; p < 0.001). A statistically significant decrease was observed only in Sequence 2 (i.e., GSH-C4-first), from baseline to the intermediate visit (p = 0.002). In the second half of the study period (i.e., from V4 to V6), Sequence 1 (i.e., HA-first) showed a trend toward lower OSDI scores (p = 0.056) that did not reach statistical significance.

Data reported in Table 2 indicate a significant improvement in the FACES score associated with GSH-C4 treatment (Control: 3.30 ± 0.86 vs. Experimental: 2.38 ± 0.19; p = 0.012). Although a statistically significant reduction was observed in Sequence 2 (i.e., GSH-C4-first) from baseline to the intermediate timepoint (p = 0.0312), only a modest decrease in the FACES score was noted in Sequence 1 (i.e., HA-first) from the intermediate to the final timepoint (p = 0.0625).

The analysis of the two PROMs did not reveal any carryover effect, as shown in Table 2.

### 3.3 Evaluation of cytokines and chemokines in tears during clinical study

As detailed in the materials and methods section of the manuscript, the concentration of different cytokines and chemokines in tears was analyzed using flow cytometry at four study visits following randomization: V1, V3 (1 month after the initial control or the experimental formulation treatment), V4 (after the 1-week wash-out), and V6 (1 month after the crossover treatment).

**FIGURE 3:**
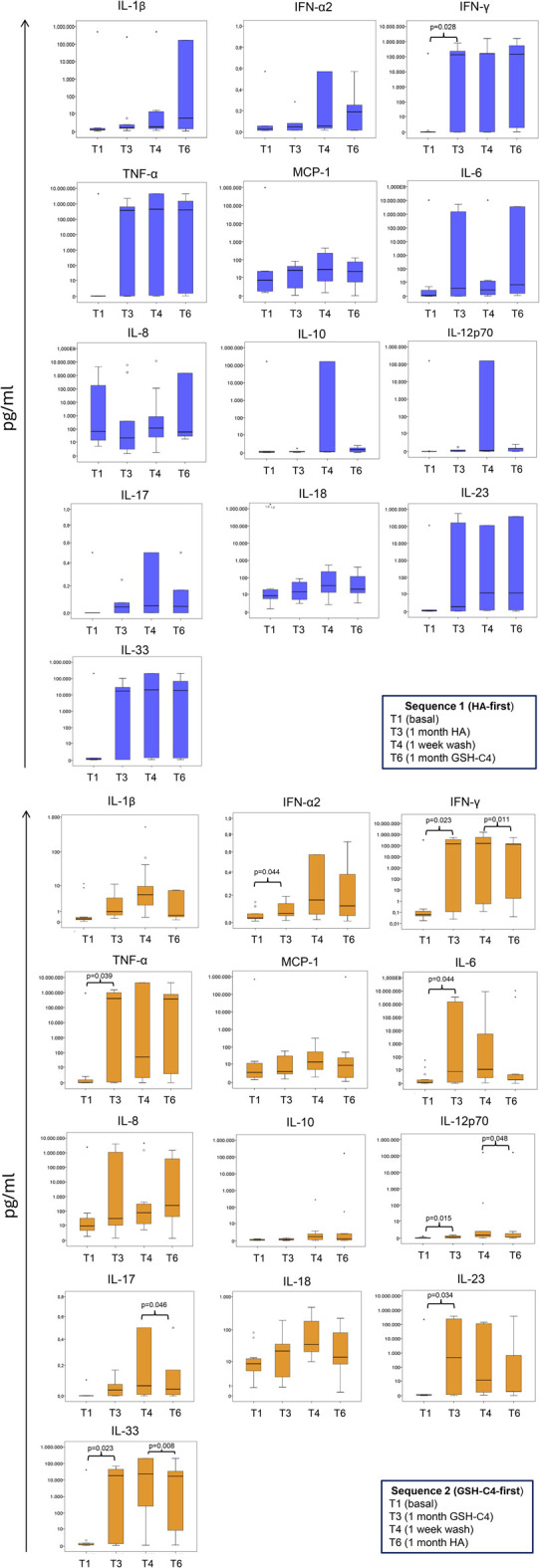
Effects of HA + GSH-C4 versus HA-only treatment on tear cytokine levels in glaucoma-associated ocular surface disease. Box plots display concentrations (pg/mL) of IL-1β, IFN-α2, IFN-γ, TNF-α, MCP-1, IL-6, IL-8, IL-10, IL-12p70, IL-17A, IL-18, IL-23, and IL-33 measured by multiplex flow cytometry (LEGENDplex™ Human Inflammation Panel 1). **(A)** Sequence 1 (HA-first): V1 = baseline, V3 = after 1 month of HA, V4 = after 1-week washout, V6 = after 1 month of HA + GSH-C4. **(B)** Sequence 2 (GSH-C4-first): V1 = baseline, V3 = after 1 month of HA + GSH-C4, V4 = after 1-week washout, V6 = after 1 month of HA. Comparisons between timepoints were performed using paired t-tests; p < 0.05 was considered statistically significant.

Cytokine dynamics are summarized in Figure 3. In the HA-first sequence (Figure 3A), after 1 month of HA-only treatment (V1 to V3), cytokine concentrations remained largely at baseline, with the sole exception of IFN-γ, which showed a modest but significant increase (p = 0.028). Following crossover to HA + GSH-C4 (V3 to V6), directional increases were observed for several mediators, although these changes did not achieve statistical significance compared to baseline, mirroring the trends seen in clinical outcomes for this sequence. By contrast, in the GSH-C4-first sequence (Figure 3B), HA + GSH-C4 elicited a reproducible cytokine activation profile: at V3, levels of IFN-α2 (p = 0.044), IFN-γ (p = 0.023), TNF-α (p = 0.039), IL-6 (p = 0.044), IL-12p70 (p = 0.015), IL-23 (p = 0.034), and the epithelial alarmin IL-33 (p = 0.023) were significantly elevated versus baseline, whereas IL-1β, MCP-1, IL-8, IL-10, and IL-18 remained unchanged. After treatment withdrawal (V4), this response contracted selectively, with IL-17A (p = 0.046) and IL-33 (p = 0.008) declining significantly, while other mediators showed partial but non-significant attenuation. At V6, after 1 month of HA-only treatment, most cytokines returned toward baseline; however, IFN-γ (p = 0.011) and IL-12p70 (p = 0.048) remained significantly elevated, consistent with a sustained Th1-polarising signal.

Together, these findings suggest that HA + GSH-C4 induces a transient, reversible cytokine activation with repair-associated Th1/Th17 features, with IFN-γ and IL-12p70 persisting even after crossover to HA alone.

### 3.4 The correlation of cytokines with clinical parameters after HA + GSH-C4 treatment *in vivo*

As shown in Table 3, Spearman’s rank correlation analysis was performed to assess the relationship between tear-fluid cytokine concentrations and clinical indices of G-OSD in the GSH-C4-first treatment arm. At baseline (V1), NEI-SS scores correlated positively with IFN-γ (ρ = 0.563; p = 0.023), IL-8 (ρ = 0.697; p = 0.003), IL-10 (ρ = 0.518; p = 0.040), IL-17A (ρ = 0.730; p = 0.003), IL-23 (ρ = 0.693; p = 0.003), and IL-33 (ρ = 0.629; p = 0.009). The FACES score showed significant positive correlations with IFN-α2 (ρ = 0.569; p = 0.022), IL-10 (ρ = 0.521; p = 0.039), IL-17A (ρ = 0.582; p = 0.029), and IL-33 (ρ = 0.530; p = 0.036). Tear film break-up time (TFBUT) was positively associated with IL-8 (ρ = 0.573; p = 0.020) and inversely with TNF-α (ρ = −0.501; p = 0.048).

**TABLE 3:** Spearman’s rank correlation coefficient (Rho) between tear‐fluid cytokine concentrations and clinical indices of glaucoma‐related ocular surface disease in the GSH-C4-first treatment arm. Correlations are shown for V1 (left columns) and after 1 month of topical experimental treatment instillation (V3, right columns). Significant correlations (p < 0.05) are shown in bold (Abbreviations. OSDI = Ocular Surface Disease Index; NEI = National Eye Institute fluorescein staining score; TFBUT = Tear Film Break-up Time; Rho = Spearman’s rank correlation coefficient.)

Cytokine (pg/mL)	​	V1					V3 (following GSH-C4 treatment)				
	​	OSDI	NEI	Faces score	Schirmer test	TBUT	OSDI	NEI	Faces score	Schirmer test	TBUT
IL-1β	Rho	−0.261	0.386	0.018	−0.094	−0.277	0.018	−0.022	**−0.626**	0.494	0.078
	p	0.347	0.155	0.948	0.739	0.318	0.948	0.935	**0.010**	0.052	0.774
IFN-α2	Rho	−0.071	0.462	**0.569**	−0.177	−0.290	0.227	−0.434	−0.072	**0.837**	0.349
	p	0.793	0.072	**0.022**	0.511	0.276	0.397	0.093	0.792	**0.000**	0.185
IFN-γ	Rho	−0.368	**0.563**	0.385	−0.394	−0.085	0.060	−0.395	−0.191	**0.741**	0.120
	p	0.161	**0.023**	0.141	0.131	0.756	0.826	0.130	0.478	**0.001**	0.657
TNF-α	Rho	−0.160	0.335	0.141	−0.009	**−0.501**	0.015	−0.438	−0.129	**0.723**	0.134
	p	0.554	0.204	0.604	0.973	**0.048**	0.956	0.089	0.635	**0.002**	0.621
MCP-1	Rho	0.030	0.135	0.049	0.458	0.337	0.065	**−0.743**	0.277	**0.726**	0.114
	p	0.913	0.618	0.858	0.075	0.201	0.810	**0.001**	0.299	**0.001**	0.675
IL-6	Rho	0.130	0.307	0.207	0.439	0.000	0.113	−0.385	−0.095	**0.770**	0.207
	p	0.631	0.247	0.441	0.089	>0.999	0.676	0.141	0.725	**0.000**	0.442
IL-8	Rho	−0.213	**0.697**	0.269	−0.046	**0.573**	−0.265	−0.280	−0.301	0.441	0.009
	p	0.428	**0.003**	0.314	0.866	**0.020**	0.321	0.294	0.257	0.087	0.974
IL-10	Rho	−0.071	**0.518**	**0.521**	−0.129	−0.393	0.096	**−0.533**	−0.036	**0.675**	0.479
	p	0.793	**0.040**	**0.039**	0.635	0.132	0.723	**0.033**	0.894	**0.004**	0.061
IL-12p70	Rho	−0.167	0.370	0.429	−0.135	−0.424	0.054	−0.454	−0.153	**0.737**	0.066
	p	0.538	0.158	0.097	0.618	0.102	0.843	0.078	0.572	**0.001**	0.807
IL-17A	Rho	−0.321	**0.730**	**0.582**	−0.430	−0.309	0.000	−0.456	−0.108	**0.723**	0.139
	p	0.262	**0.003**	**0.029**	0.125	0.282	>0.999	0.076	0.689	**0.002**	0.607
IL-18	Rho	−0.325	0.375	0.183	−0.040	0.690	0.071	−0.114	−0.143	**0.607**	−0.168
	p	0.219	0.153	0.497	0.884	0.799	0.793	0.674	0.598	**0.013**	0.535
IL-23	Rho	−0.131	**0.693**	0.389	−0.135	−0.160	0.015	−0.438	−0.129	**0.723**	0.134
	p	0.630	**0.003**	0.137	0.619	0.554	0.956	0.089	0.000	**0.002**	0.621
IL-33	Rho	−0.232	**0.629**	**0.530**	−0.349	−0.206	0.030	−0.425	−0.150	0.732	0.130
	p	0.388	**0.009**	**0.035**	0.185	0.445	0.912	0.101	0.579	0.001	0.632

The numbers in bold are statistically significant.

After 1 month of HA + GSH-C4 treatment (V3), NEI-SS exhibited significant negative correlations with MCP-1 (ρ = −0.743; p = 0.001) and IL-10 (ρ = −0.533; p = 0.033), while the FACES score correlated negatively with IL-1β (ρ = −0.626; p = 0.010). In contrast, Schirmer I test values showed significant positive correlations with a broad panel of cytokines, including IFN-α2 (ρ = 0.837; p < 0.001), IFN-γ (ρ = 0.741; p = 0.001), TNF-α (ρ = 0.723; p = 0.002), MCP-1 (ρ = 0.726; p = 0.001), IL-6 (ρ = 0.770; p < 0.001), IL-10 (ρ = 0.675; p = 0.004), IL-12p70 (ρ = 0.737; p = 0.001), IL-17A (ρ = 0.723; p = 0.002), IL-18 (ρ = 0.607; p = 0.017), and IL-23 (ρ = 0.723; p = 0.002). No significant correlations were observed for TFBUT at this time point. These findings highlight a shift from a baseline inflammatory profile associated with ocular surface damage toward a post-treatment milieu in which cytokine expression parallels improvements in tear production and PROMs.

### 3.5 The GSH-c4-containing eye drop formulation promotes *in vitro* damage repair under stress conditions

**FIGURE 4:**
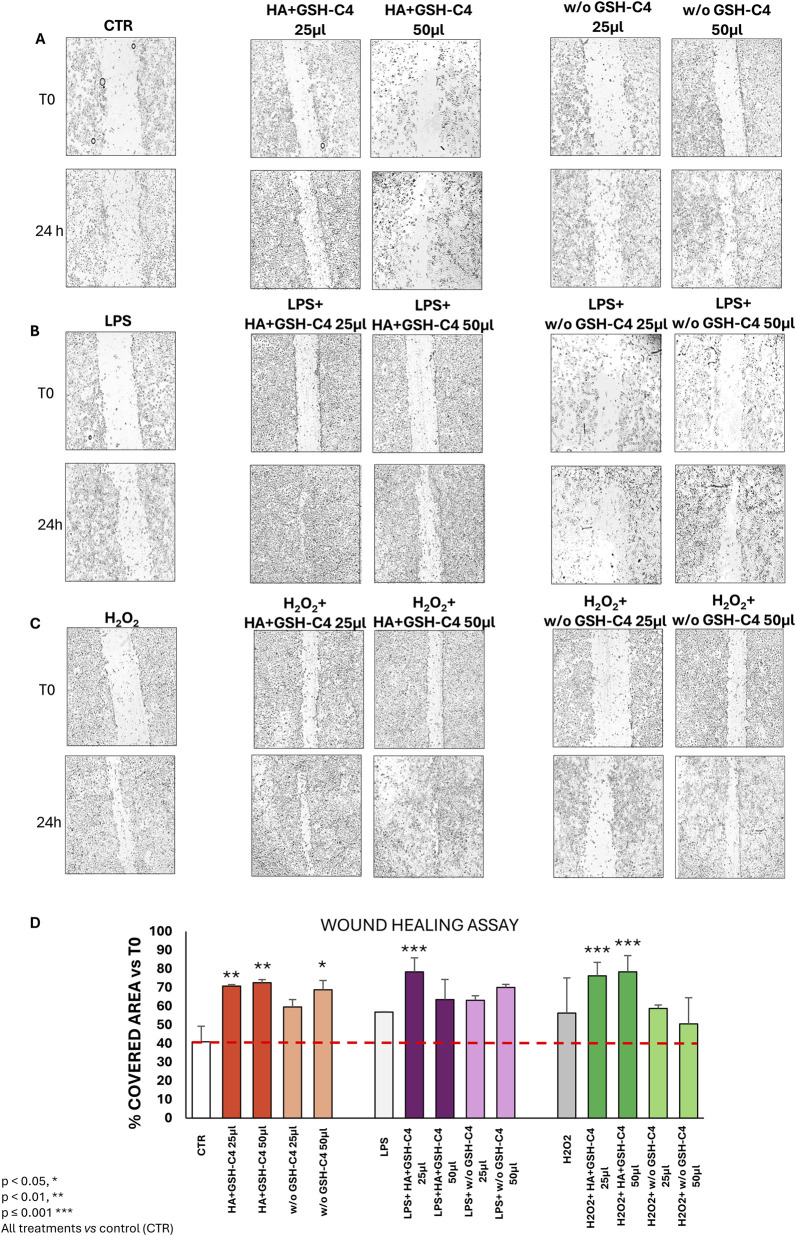
Evaluation of corneal epithelial cell migration *in vitro*. A wound-healing assay was performed in human corneal epithelial cells (HCEpiC) to assess the effects of HA + GSH-C4 (25 μL, 50 µL) and HA-only (25 μL, 50 µL), under basal conditions **(A)** and in stress environments induced by LPS 1 μg/mL **(B)** or H_2_O_2_ 25 µM **(C)**. Representative images of the scratch area are shown at baseline (T0) and after 24 h. Data are presented as mean ± standard deviation (SD) of at least three independent experiments. One-way analysis of variance (ANOVA) with Bonferroni post-hoc test was used for comparisons (*p < 0.05; **p < 0.01; ***p ≤ 0.001). Black asterisks denote significance relative to Controls (CTR) **(D)**.

To evaluate the reparative capacity of HA + GSH-C4 on corneal epithelial injury, we conducted an *in vitro* wound-healing assay using HCEpiC (Figure 4). Under basal growth conditions (Figure 4A), HA + GSH-C4 significantly accelerated scratch closure at both 25 μL and 50 µL doses compared with control (p = 0.009 and p = 0.004, respectively), whereas the HA-only formulation produced a comparable effect only at 50 µL (p = 0.022). In an LPS-induced inflammatory environment (Figure 4B), enhanced wound closure was observed exclusively with HA + GSH-C4 at 25 µL (p < 0.001), with no significant benefit from other treatments. Under oxidative stress induced by H_2_O_2_ (Figure 4C), both 25 μL and 50 µL doses of HA + GSH-C4 promoted significantly greater wound repair compared with control (p = 0.001 and p < 0.001, respectively). Quantitative analysis of wound closure at 24 h is presented in Figure 4D; Table 4.

**TABLE 4:** Evaluation of cell migration. Analysis of wound closure in different experimental conditions calculated using ImageJ software.

*in vitro treatments*	*% covered area (24h* vs*. T0)± SD*
CTR	40.89 ± 8.37
HA + GSH-C4 25 μL	**70,73** ^**^ ± 0.81
HA + GSH-C4 50 μL	**72,55** ^**^ ± 1.67
W/o GSH-C4 25 μL	59.56 ± 3.95
W/o GSH-C4 50 μL	**68,77** ^*^ ± 4.98
LPS	56.80 ± 0.23
LPS + HA + GSH-C4 25 μL	**78,36** ^***^ ± 7.50
LPS + HA + GSH-C4 50 μL	63.49 ± 10.81
LPS + w/o GSH-C4 25 μL	63.13 ± 2.42
LPS + w/o GSH-C4 50 μL	70.00 ± 1.66
H2O2	56.27 ± 18.85
H2O2 + HA + GSH-C4 25 μL	**76,23** ^***^ ± 7.25
H2O2 + HA + GSH-C4 50 μL	**78,37** ^***^ ± 8.70
H2O2 + w/o GSH-C4 25 μL	58.74 ± 1.85
H2O2 + w/o GSH-C4 50 μL	50.49 ± 14.02

ANOVA test with Bonferroni post-hoc multiple comparison was performed (*p ≤ 0.050, **p < 0.010, ***p ≤ 0.001) and “*” reports significance with respect to CTR. Data report the mean ± the Standard Deviation (SD) of at least three experiments.

The numbers in bold are statistically significant.

### 3.6 Evaluation of cytokines modulation during wound healing *in vitro* model

To parallel the *in vivo* analyses, soluble mediator levels were measured in supernatants from HCEpiC scratch-wound assays treated with HA + GSH-C4 or HA-only, under basal conditions and following LPS- or H_2_O_2_-induced stress (Supplementary Figure S1). Of the 13 cytokines assayed, epithelial cells secreted detectable amounts of IL-1β, IFN-α2, MCP-1, IL-6, IL-8, and IL-10, with treatment-dependent modulation observed only in MCP-1, IL-6, and IL-10 (Table 5). Across all conditions, HA + GSH-C4 consistently suppressed MCP-1 relative to control. Under LPS challenge, MCP-1 was significantly reduced by both 25 μL and 50 µL doses of HA + GSH-C4 compared with LPS alone (p = 0.009 and p = 0.001, respectively). A similar pattern emerged under oxidative stress: all formulations lowered MCP-1, but inhibition was most pronounced with HA + GSH-C4 (all p ≤ 0.001). By contrast, HA + GSH-C4 did not significantly alter IL-6 or IL-10, whereas the HA-only preparation at 50 µL induced a modest reduction in both cytokines during H_2_O_2_ exposure (p ≤ 0.006). Collectively, these data indicate that HA + GSH-C4 selectively and robustly attenuates MCP-1 secretion, while effects on IL-6 and IL-10 are limited to high-dose HA-only treatment under oxidative stress.

**TABLE 5:** Evaluation of supernatant-derived cytokines after *in vitro* treatments. Cytokine levels (pg/mL) from supernatant of HCEpiC cell line treated with HA + GSH-C4 and w/o GSH-C4 (25µL/well and 50µL/well) both in the absence and presence of LPS (1ug/mL) or H2O2 (25uM). Expression levels of MCP-1 (CCL2), IL-6, IL-10, IL-1β, IFN-α2, IL-8 were analyzed by flow cytometry using LEGENDplex^™^ Human Inflammation Panel 1, and represented as table. Analysis of cytokine levels in different experimental conditions calculated using SPSS software.

*in vitro treatments*	*Cytokines (pg/mL) ± SD*					
	MCP-1	IL-6	IL-10	IL-1β	IFN-α2	IL-8
CTR	421.48 ± 73.02	2127.86 ± 657.99	768.41 ± 536.17	25.76 ± 12.05	2.78 ± 0.63	153.5 ± 208.95
HA + GSH-C4 25 μL	**158,62** ^***^ ± 36.25	2012.27 ± 639.49	837.45 ± 312.08	23.04 ± 9.70	3.10 ± 0.30	61.13 ± 14.78
HA + GSH-C4 50 μL	**135,38** ^***^ ± 13.59	2015.46 ± 240.37	1035.44 ± 279.80	28.76 ± 4.63	3.01 ± 0.75	63.24 ± 26.67
W/o GSH-C4 25 μL	**209,25** ^**^ ± 50.72	1858.92 ± 451.15	576.80 ± 405.04	29.26 ± 13.93	3.03 ± 0.46	393.65 ± 710.66
W/o GSH-C4 50 μL	256.38 ± 101.84	2534.4 ± 775.14	1251.44 ± 155.15	33.46 ± 9.91	3.26 ± 0.75	29.33 ± 12.62
LPS	547.11 ± 142.94	2495.02 ± 189.25	1073.71 ± 254.73	30.26 ± 3.35	2.98 ± 0.67	21.26 ± 11.46
LPS + HA + GSH-C4 25 μL	**334,02** ± 100.68	1966.72 ± 138.46	1060.76 ± 226.09	22.82 ± 6.55	3.00 ± 0.68	30.21 ± 18.39
LPS + HA + GSH-C4 50 μL	**306,81** ± 62.42	2082.42 ± 791.12	1284.16 ± 541.03	31.57 ± 6.11	2.97 ± 0.62	29.27 ± 20.56
LPS + w/o GSH-C4 25 μL	377.47 ± 62.86	2538.82 ± 667.62	1567.04 ± 524.53	31.08 ± 12.82	3.05 ± 0.65	17.86 ± 6.53
LPS + w/o GSH-C4 50 μL	357.87 ± 21.56	2510.13 ± 513.58	1358.17 ± 288.19	28.02 ± 4.61	3.18 ± 0.71	23.23 ± 20.82
H2O2	257.85 ± 49.31	1879.35 ± 928.42	567.56 ± 371.99	36.05 ± 4.43	2.69 ± 0.30	323.45 ± 527.22
H2O2 +HA + GSH-C4 25 μL	**94,16** ^***^ ± 23.00	961.9 ± 409.80	221.68 ± 365.45	29.23 ± 4.67	2.85 ± 0.46	583.31 ± 557.87
H2O2 +HA + GSH-C4 50 μL	**113,01** ^***^ ± 16.15	1521.37 ± 682.40	734.48 ± 613.56	30.96 ± 4.51	2.94 ± 0.60	412 ± 700.25
H2O2 + w/o GSH-C4 25 μL	**179,70** ^**^ ± 75.04	1510.60 ± 730.17	592.10 ± 419.31	27.67 ± 10.12	2.75 ± 0.60	259.05 ± 366.69
H2O2 + w/o GSH-C4 50 μL	**57,61** ^***^ ± 50.70	**461,48** ^**##**^ ± 390.31	**5,97** ^**##**^ ± 4.95	20.98 ± 4.04	3.51 ± 0.35	642.53 ± 312.13

ANOVA test with Bonferroni post-hoc multiple comparison was performed (*p ≤ 0.050, **p < 0.010, ***p ≤ 0.001) and “*” reports significance with respect to CTR; “°” reports significance with respect to LPS; “^#^” reports significance with respect to w/o C4 (50 μL). Data report the mean ± the Standard Deviation (SD) of at least three experiments.

The numbers in bold are statistically significant.

## 4 Discussion

In this preliminary, hypothesis-generating, prospective, double-blind, cross-over interventional study, we examined efficacy and mechanistic effects of a novel tear substitute containing 0.2% HA acid supplemented with 0.4% butyroyl-glutathione (i.e., GSH-C4) in patients with G-OSD. The GSH-C4-enriched formulation yielded clinically relevant improvements: patient-reported symptoms improved significantly (lower OSDI and Faces scores), tear-film stability increased (longer TFBUT), and corneal staining decreased (lower NEI-SS score). Tear volume, as measured by the Schirmer test, remained unchanged, indicating that symptom relief was driven primarily by improved film quality rather than augmented aqueous production. The change in group-level Schirmer values reached a borderline significant p-value (p = 0.054), however, considering the small sample size, the significant correlation with multiple cytokines at the V3 time point supports the efficacy of the combined treatment with GSH-C4. Altogether, these results suggest effects at the epithelial/mucin–lipid interface rather than a primary lacrimal-secretagogue mechanism. Cytokine profiling revealed that only HA + GSH-C4 elicited significant modulation of tear mediators, and post-treatment cytokine levels correlated directly with other clinical metrics, consistent with a glutathione-mediated rebalancing of the ocular surface microenvironment. These clinical and molecular findings were paralleled by *in vitro* wound-healing assays, in which HA + GSH-C4 significantly accelerated epithelial repair under basal, inflammatory, and oxidative conditions, further supporting its protective and reparative potential. Collectively, these results support HA + GSH-C4–fortified lubricants as a promising therapeutic strategy for G-OSD.

The observed improvement in tear film stability, as measured by TFBUT, is particularly relevant given G-OSD is predominantly associated with Meibomian gland dysfunction (MGD), leading to an evaporative form of DED characterized by poor tear film stability (Nijm et al., 2020; Wellik and Galor, 2023). In fact, prostaglandin analogues, pilocarpine, and timolol have been shown to induce obstructive MGD by directly affecting Meibomian cells and influencing their morphology, survival, and proliferative capacity (Mocan et al., 2016; Zhang et al., 2017; Ha et al., 2019; Sharma et al., 2018). Given the presence of glutathione peroxidase in meibomian glands and conjunctiva and its dependence on intracellular GSH, augmenting ocular surface redox capacity with GSH-C4 may mitigate ROS-driven epithelial stress and thereby support tear-film stability, a hypothesis-generating observation that warrants further study (Nezzar et al., 2017).

In our cohort, glutathione use was associated with reduced ocular surface damage, as indicated by NEI-SS scores. Antiglaucoma drops - particularly those containing benzalkonium chloride - have been directly linked to ocular surface damage (Mocan et al., 2016; Zhang et al., 2017; Ha et al., 2019; Sharma et al., 2018). Several mechanisms have been described to contribute to cellular death in G-OSD. Sedlak et al. reported that prostaglandin F2α analogues induce inflammation and local oxidative stress in patients with ocular hypertension or glaucoma, including reduced glutathione peroxidase activity (Sedlak et al., 2019). Tovar et al. observed that eyes receiving topical hypotensive medications exhibit significantly higher concentrations of caspase-1, a key inflammasome mediator involved in DED, with levels correlating to both the number of medications used and ocular surface damage (Tovar et al., 2022). Caspase-1 drives inflammation by releasing cytokines such as IL-1β and IL-18, causing cell lysis and attracting immune cells (Adamczak et al., 2014; Baroja-Mazo et al., 2014). This initiates a cycle of inflammation that persists until the underlying insult is resolved (Adamczak et al., 2014; Baroja-Mazo et al., 2014). Because glutathione peroxidase activity counters caspase-1 activation, supplementing with glutathione may help mitigate this inflammatory pathway by reducing local oxidative stress and inflammation, ultimately decreasing damage to both cellular and extracellular structures on the ocular surface (Circu and Aw, 2012; Sedlak et al., 2021).

Beyond their well-recognized lubricating properties, both HA and glutathione-based compounds exert biologically relevant effects on the ocular surface that may contribute to the observed clinical and molecular findings. HA has been shown to modulate ocular surface inflammation through interactions with CD44 and other cell-surface receptors, thereby influencing leukocyte trafficking and reducing the expression of pro-inflammatory cytokines and chemokines (Oh et al., 2014). In parallel, accumulating evidence indicates that oxidative stress plays a central role in ocular surface disease, with excessive ROS contributing to epithelial damage, immune activation, and impaired healing (Wu et al., 2024). In other words, GSH represents a key endogenous antioxidant, acting as a major cellular defence system through direct scavenging of free radicals and regulation of redox-sensitive signalling pathways (Nezzar et al., 2017; Wu et al., 2024). Restoration of the oxidant–antioxidant balance has been shown to mitigate cellular dysfunction and inflammation in corneal tissues, highlighting the therapeutic relevance of GSH and related compounds.

One of the most intriguingly and seemingly counterintuitive findings of our study was the post‐treatment surge in tear‐fluid cytokines (IFN-α2, IFN-γ, TNF-α, IL-6, IL-23 and IL-33) that accompanied clinical improvements in tear‐film stability and corneal integrity. Our two‐period, double‐blind crossover design permitted a longitudinal profiling of cytokine trajectories across four visits, in marked contrast to cross‐sectional investigations that provide only a single snapshot of ocular inflammation. Notably, cytokine levels at V1 did not differ between sequences, indicating a comparable subclinical inflammatory milieu. HA + GSH-C4 exposure was associated with transient elevations in selected mediators (e.g., IFN-α2, IFN-γ, TNF-α, IL-6, IL-23, IL-33), whereas HA-only produced minimal changes. After wash-out and crossover, increases were again most evident when HA + GSH-C4 was introduced; in the HA-first sequence these changes were directionally similar but not statistically significant. Taken together, the pattern suggests that a controlled, short-lived immune activation may accompany epithelial repair, consistent with the accelerated wound closure observed in HCEpiC assays under basal, inflammatory and oxidative stress conditions (Behm et al., 2012).

Importantly, the interpretation of increased Th1-associated cytokines as part of a repair-associated response rather than purely deleterious inflammation is supported by emerging evidence on corneal wound healing biology (Wilson, 2020). Experimental and translational studies indicate that acute activation of the innate immune system is an essential and tightly regulated component of epithelial repair, facilitating debris clearance, antimicrobial defence, and activation of migratory and proliferative pathways in corneal epithelial cells (Fortingo et al., 2022). In this context, cytokines traditionally classified as “pro-inflammatory”, including IFN-γ and TNF-α, can contribute to early wound-healing phases by promoting immune cell recruitment, epithelial–immune crosstalk, and growth factor signalling cascades, ultimately supporting re-epithelialization (Aiello et al., 2020). Conversely, it is the persistence or dysregulation of these pathways (rather than their transient activation) that is associated with impaired healing and tissue damage (Lin et al., 2025). Consistent with this paradigm, recent single-cell analyses of the corneal immune microenvironment have demonstrated that immune activation during physiological wound healing is characterized by dynamic and reversible shifts in immune cell phenotypes, whereas chronic or exaggerated responses, such as those observed in diabetic corneas, lead to maladaptive inflammation and delayed repair (Lin et al., 2025). Therefore, the transient cytokine elevation observed in our study likely reflects a controlled, context-dependent immune activation that accompanies epithelial repair, rather than a sustained pro-inflammatory state.

It is also worth mentioning that cellular redox homeostasis could influence cellular pathways influencing cell fate and differentiation (Ye et al., 2015). Among the mediators modulated by HA + GSH-C4 is IL-33, a prototypical “alarmin” released upon epithelial damage that has been shown to expand and activate corneal group 2 innate lymphoid cells (ILC2), thereby accelerating tissue repair (Liu et al., 2017; Qian and Zhang, 2020). Through its cognate receptor ST2, the IL-33/ST2 axis further orchestrates diverse innate immune populations and polarizes macrophages toward a reparative phenotype, a mechanism well characterized in cutaneous wound healing (Yin et al., 2013). At the same time, it should be acknowledged that IL-33 can also amplify type-2 responses in other contexts, underscoring the importance of dose, timing, and tissue state in determining its net effect (Behm et al., 2012; Wilson, 2020; Fortingo et al., 2022).

Among the strengths of the present study, the prospective, double-blind, two-period crossover design enabled within-subject assessment of clinical outcomes and tear-fluid cytokines, minimizing inter-individual variability and improving efficiency to detect reversible changes. Our priori calculation—based on TNF-α variance—ensured adequate sensitivity for the primary powered biomarker, while clinical endpoints were exploratory and likely underpowered. No treatment-related adverse events or tolerability issues were observed; nevertheless, longer exposure is needed to assess chronic safety. The inclusion of patients on diverse regimens, both preserved and preservative-free, supports the generalizability of the effects of the formulation across a representative spectrum of real-world therapy. Finally, integration of *in vitro* scratch-wound assays under basal, inflammatory (LPS), and oxidative (H_2_O_2_) stress conditions provides mechanistic corroboration that HA + GSH-C4 may exert a direct pro-repair effect on corneal epithelial cells.

Several factors temper the interpretation and generalizability of our findings. First, the small cohort (16 eyes randomized, 15 analyzable) was adequately powered for the primary TNF-α endpoint but may be underpowered for secondary outcomes, thereby limiting statistical reliability for these measures. Although TFBUT was the primary clinical endpoint, the study was powered on TNF-α as a biologically relevant surrogate due to limited prior ophthalmic data for GSH-C4; clinical endpoints are therefore hypothesis-generating. In addition, given the number of cytokines and timepoint comparisons, our biomarker analyses are subject to multiplicity and were not adjusted for multiple comparisons; findings should therefore be interpreted as exploratory. Second, heterogeneity in topical hypotensive therapy (various drug classes and preservative status) introduces uncontrolled confounding; the study was neither stratified nor powered to dissect differential effects by medication type, dosing regimen, or preservative load. Third, the one-week washout interval, although sufficient to reverse most clinical effects, may not completely eliminate slower-resolving mediators, potentially attenuating period‐to‐period contrasts. Fourth, our focus on G-OSD constrains applicability to other dry-eye etiologies (e.g., post‐surgical, autoimmune) and non-glaucomatous populations. Fifth, the short duration of each treatment phase (1 month) and absence of long-term follow-up preclude conclusions about sustained efficacy, safety, and adherence.

In addition, the *in vitro* assays are exploratory by design. The wide confidence intervals observed for cytokine modulation reflect substantial biological variability and caution against overinterpretation. Moreover, these experiments do not establish causality between HA + GSH-C4 exposure and repair pathways. Finally, although our flow cytometry panel comprehensively quantified key tear‐fluid mediators, it was not designed to delineate the intracellular signaling cascades or upstream regulators driving G-OSD which herein appears as a multifaceted clinical condition. Ultimately, randomized multicenter trials with extended follow-up will be required to confirm these findings and establish the long-term safety and efficacy of HA + GSH-C4 in G-OSD.

In conclusion, the results from this preliminary crossover study suggest that the combination of hyaluronic acid and GSH-C4 is associated with improvements in symptoms and tear-film stability, together with reduced corneal staining in G-OSD, without altering tear volume. Treatment-specific, transient elevations in selected cytokines, together with accelerated epithelial wound healing *in vitro*, support a dual mode of action: barrier stabilization coupled with an adaptive pro-repair immune response. Future studies should confirm these outcomes in larger, stratified cohorts, assess long-term effects, and delineate the immunomodulatory molecular pathways through which GSH-C4 influences the ocular surface.

## Data Availability

The raw data supporting the conclusions of this article will be made available by the authors, without undue reservation.
