# Supplementary figures and images for: Efficacy of hyaluronic acid and butyroyl glutathione in the management of glaucoma-related ocular surface disease: a prospective, interventional, double-blind, cross-over post market study
Source: Front Pharmacol. 2026 Jun 5;17:1780815. doi: 10.3389/fphar.2026.1780815 (PMC13279420; doi:10.3389/fphar.2026.1780815)

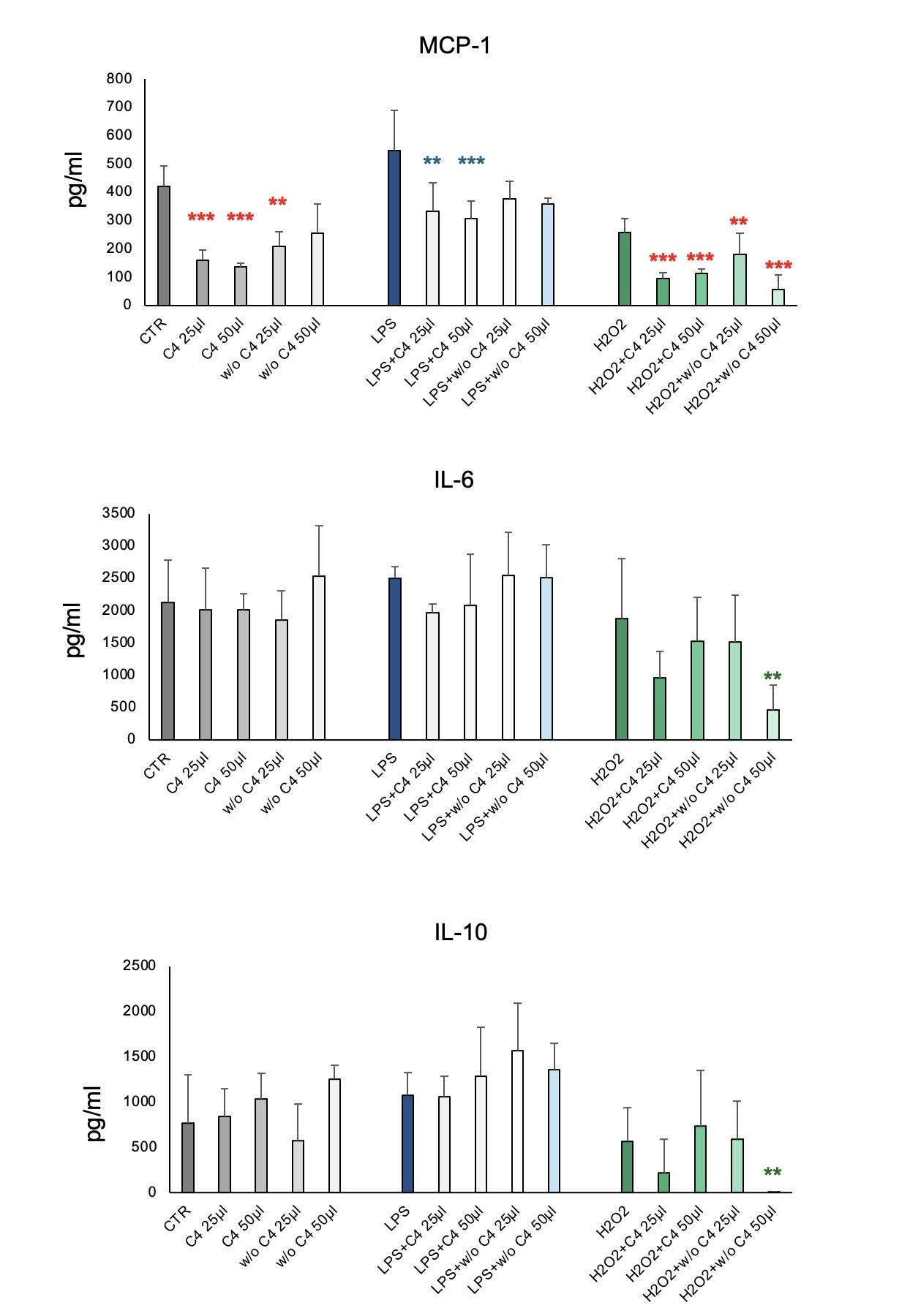

Supplement: Supplementary file 1 [file image1.jpg]
